# Causal relationships of physical activity and leisure sedentary behaviors with scoliosis: A two-sample Mendelian randomization study

**DOI:** 10.1097/MD.0000000000051514

**Published:** 2026-11-06

**Authors:** Fangjun Yang, Xiaoming Qiu, Han Liu, Jiantao Wen

**Affiliations:** a Department of Orthopaedics, Gansu Provincial Hospital of Traditional Chinese Medicine, Lanzhou, China; b Gansu Provincial Hospital of Traditional Chinese Medicine (The First Affiliated Hospital of Gansu University of Traditional Chinese Medicine) Gansu University of Traditional Chinese Medicine, Lanzhou, China; c Department of Medical Engineering, The First Affiliated Hospital of Army Medical University, Chongqing, China; d Department of Spinal Orthopedics, Gansu Provincial Hospital of Traditional Chinese Medicine, Lanzhou, China.

**Keywords:** causality, leisure sedentary behavior, Mendelian randomization, physical activity, scoliosis

## Abstract

Scoliosis is the most common spinal deformity in children and adolescents, and modifiable lifestyle factors such as sedentary behavior and physical activity (PA) have been suggested to influence its risk, although evidence remains conflicting. This study aimed to investigate the relationships between leisure sedentary behaviors, PA, and the risk of scoliosis by using two-sample Mendelian randomization (MR). Genetic variation in television watching time, mobile phone use time, moderate-to-high intensity physical activity (MVPA), and vigorous physical activity (VPA) were used as IVs. The outcome data were derived from the Finn-Gen Scoliosis dataset. The causal relationships were estimated with inverse variance weighted (IVW), MR-Egger, and weighted median methods. Sensitivity analysis was conducted using Cochran *Q* test, MR-Egger intercept test, Mendelian Randomization Pleiotropy Residual Sum and Outlier (MR-PRESSO), leave-one-out analysis, and funnel plot to estimate the robustness of our findings. The IVW results indicated the following: watching TV time (odds ration [OR] = 0.623, 95% confidence interval [CI]: 0.21–1.78, *P* = .38); time spent on mobile phones (OR = 0.57, 95% CI: 0.16–1.96, *P* = .37); MVPA (OR = 0.53, 95% CI: 0.09–3.15, *P* = .49); VPA (OR = 0.62, 95% CI: 0.21–1.78, *P* = .61). The primary IVW MR analysis results were consistent with those of the multiple sensitivity MR Analysis. Genetic susceptibility to leisure sedentary behavior and PA are not associated with the risk of scoliosis. The observed associations may be the result of shared genetic effects or environmental confounding factors.

## 1. Introduction

Scoliosis is defined as a lateral curvature of the spine that is 10 degrees or greater on a coronal radiographic image, representing a three-dimensional deformity.^[1,2]^ As the most common clinical spinal malformation, scoliosis affects 3% of children worldwide, with over 80% of cases being idiopathic.^[1,3]^ The etiology of scoliosis remains largely unknown but is thought to involve genetic, metabolic, biomechanical, and environmental factors.^[4–10]^ Leisure sedentary behavior refers to physical behavior that relies on lying down and sitting to maintain a low metabolic state (energy consumption ≤ 1.5 metabolic equivalent), including watching TV and using mobile phones.^[11]^ Physical activity (PA) is defined as any bodily movement produced by skeletal muscles that requires energy expenditure.^[12]^ Sedentary behavior has been linked to a number of diseases, including cardiovascular disease, high blood pressure, diabetes, and musculoskeletal disorders.^[13–15]^ Previous studies have found that sitting not only increases the risk of these diseases and increases mortality, but PA reduces the risk.^[16]^ More than 5 million deaths could be prevented each year by ensuring adequate levels of PA.^[17,18]^ Scoliosis has no obvious symptoms in the early stage, making it easy to overlook. If not detected and treated promptly, it can lead to negative body image, psychosocial disorders, and in serious cases, compression of the spinal cord, and impairment of heart and lung function.^[19,20]^

Identifying modifiable risk factors associated with scoliosis is crucial for early intervention and prevention. Numerous studies have been conducted to explore the association between leisure sedentary behavior, PA, and scoliosis, but these studies have produced conflicting results. Some studies have found no link,^[21,22]^ while others have reported either higher^[21–24]^ or lower^[25,26]^ levels of PA in individuals with scoliosis. The reasons for these discrepancies may stem from limitations inherent in observational studies, such as confounding factors and inaccuracies in self-reported data.

To better assess the potential causal relationship, we applied Mendelian randomization (MR). MR is an epidemiological method that uses genetic variants, such as single nucleotide polymorphisms (SNPs), as instrumental variables (IVs) to infer causality while minimizing confounding.^[27–29]^ In this study, we used television watching time, mobile phone use time, moderate-to-vigorous physical activity (MVPA), and vigorous physical activity (VPA) as exposures, with scoliosis as the outcome, to investigate the causal effects of these behaviors on scoliosis risk. Clarifying whether these lifestyle behaviors are causally related to scoliosis may help distinguish modifiable prevention targets from associations driven by confounding or reverse causation. This distinction has implications for school screening, lifestyle counseling, and the design of future intervention studies.

## 2. Methods

### 2.1. MR design

The study design follows the 3 basic principles of MR Research (Fig. 1). The IV is associated with exposure (assumption 1), The IV is independent of the combined effects of all confounders (assumption 2), and The IV affects the outcome only through the exposure, not via alternative pathways (assumption of independence from confounding and no horizontal pleiotropy).

**Figure 1.**
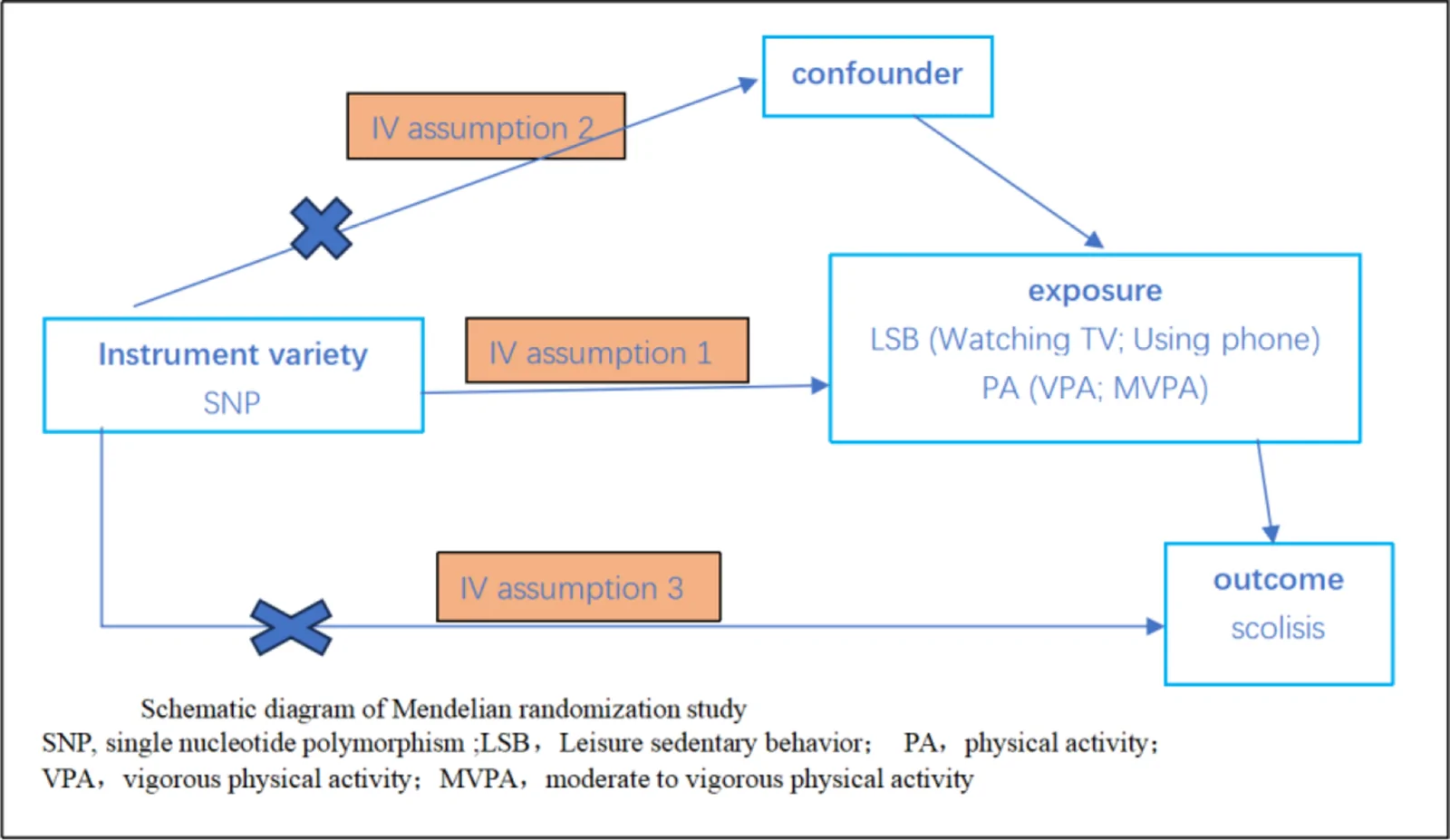
Schematic diagram of Mendelian randomization study.

### 2.2. Data source

#### 2.2.1. Exposure

TV watching time, mobile phone use time, MVPA, vigorous physical exercise (VPA). The above data were obtained from IEU Open genome-wide association study (GWAS). Ethical approval and informed consent were obtained in all original studies. The current analysis used publicly available summary statistics and was exempt from additional ethical review. The writing process of the article followed the requirements of Strengthening the Reporting of Observational Studies in Epidemiology using MR, the reporting specification for MR studies; data details are shown in Table 1.

**Table 1:** Detailed information of the studies included in the MR analyses.

GWAS ID	Year	Trait	Consortium/Author	Sample size	Number of SNPs
ukb-b-5192	2018	Time spent watching television (TV)	MRC-IEU	437,887	9,851,867
ukb-b-4094	2018	Length of mobile phone use	MRC-IEU	456,972	9,851,867
ebi-a-GCST006097	2018	Moderate-to-vigorous physical activity levels	NA	377,234	11,808,007
ebi-a-GCST006098	2018	Vigorous physical activity	NA	261,055	11,803,978

GWAS = genome-wide association study, MR = Mendelian randomization, SNP = single nucleotide polymorphism.

#### 2.2.2. Outcome (scoliosis)

Genetic statistics for scoliosis were derived from FinnGen Consortium R5 published in 2021. The large GWAS for the Finns included 1168 cases and 164,682 controls, yielding 16,380,270 SNPs for analysis after adjustment for age, sex, and genotyping batch.

### 2.3. Selection of instrumental variables (IVs)and data harmonization

In this study, SNPs meeting the following criteria were selected as IV.^[27–29]^ SNPs (*P* < 5 × 10^8^), To avoid linkage disequilibrium (LD) caused by SNPs associated with exposure, LD must satisfy *r*^2^ < 0.001, kb = 10,000, and To ensure a strong relationship between IV and exposure, we chose an *F*-statistic > 10 to avoid bias caused by weak IVs. The *F*-statistic = (β/SE)2.^[30]^ To ensure the second MR hypothesis, that genetic variants were independent of potential confounders, we conducted queries in the PhenoScanner database to exclude included IVs associated with known confounders.^[31–37]^ (Body mass index; bone mineral density; 25-hydroxyvitamin D and serum calcium; leptin; adiponectin; menstrual status and timing of puberty).

Subsequently, harmonization of the exposure and outcome summary data was performed. First, datasets were merged based on SNP rsIDs to identify overlapping SNPs. Allele alignment was then conducted for each SNP: For non-palindromic SNPs, alleles were compared directly, and the beta coefficient for the outcome was reversed if a strand flip was necessary to achieve alignment. For palindromic SNPs (i.e., A/T or C/G pairs), strand orientation was inferred by comparing the effect allele frequency (EAF). A SNP was considered matched if the absolute EAF difference between exposure and outcome datasets was <0.08; if the absolute difference between the exposure EAF and (1 – outcome EAF) was <0.08, the outcome beta was reversed. Palindromic SNPs that could not be unambiguously aligned or had missing EAF information were excluded. Finally, we confirmed that the effect alleles for all SNPs were coded consistently with respect to the exposure trait direction.

### 2.4. MR statistics and analysis

We performed a two-sample MR Analysis to estimate the causal relationship between leisure sedentary behavior, PA, and scoliosis. MR analysis methods include inverse variance weighting (IVW), weighted median (WM), simple median, weighted median estimator, and MR-Egger regression.^[38–40]^ IVW was used to calculate the odds ratio (OR) as the main method, and the slope of the weighted regression of the outcome effect on the exposure effect represented the outcome estimate (with an intercept restricted to zero). In addition, the other 4 methods were used as tests for the robustness of the primary outcome.

Mendelian Randomization Pleiotropy Residual Sum and Outlier (MR-PRESSO) is a sensitivity analysis used to test and calibrate outliers for horizontal pleiotropy, removing outliers from IVs.^[38]^ Heterogeneity of individual estimates of genetic variation was assessed using Cochran *Q* test. A *P* value of Cochran *Q* test > .05 indicated that there was no significant heterogeneity among the SNPs.^[41]^ MR-Egger regression was used to account for horizontal pleiotropy, and the absence of horizontal pleiotropy was indicated when the *P*-value of the intercept was >.05.^[42]^ Horizontal pleiotropy was tested by drawing a funnel plot. If the funnel plot shows a symmetric shape, this usually means that there is no obvious pleiotropy. Finally, leave-one-out sensitivity analyses were performed to confirm the stability and accuracy of the MR results and to evaluate SNPs that may have significant effects.

All statistical analyses were performed using R Studio (version 4.3.1), two-sample MR (version 0.5.7), and MR-PRESSO (version 1.0) software packages.^[29]^ Results are presented as ORs with 95% confidence intervals (95% CI); *P* < .05 was considered statistically significant.

## 3. Results

We first selected and harmonized genetic instruments for each exposure and then performed two-sample MR using IVW as the primary method, supplemented by sensitivity analyses. The final number of SNPs and the complete MR estimates are presented in Table 2. After considering LD effects, the possible presence of palindromic SNPs and weak IVs was excluded. Key SNP data were further collected, including effect alleles, beta, standard errors, and *P*-values, and each SNP showed sufficient validity with an *F*-value >10.^[43]^ The final set of SNPs used for the MR analysis for each exposure is presented in Table 2.

**Table 2:** Results of Mendelian randomization analysis of the 2 samples.

		Exposures			
		Watching-TV	Use the mobile phone	MVPA	VPA
SNPs (n)		64	24	12	5
IVW	OR	0.623	0.57	0.53	0.62
	95% CI	0.21–1.78	0.16–1.96	0.09–3.15	0.21–1.78
	*P* value	.38	.37	.490	.61
Weighted median	OR	0.88	0.62	0.43	0.98
	95% CI	0.20–3.83	0.03–10.96	0.03–5.30	0.002–9.94
	*P* value	.87	.75	.51	.99
Weighted mode	OR	0.88	0.59	0.24	2.73
	95% CI	0.03–24.30	0.12–2.97	0.01–11.80	0.001–2.74
	*P* value	.94	.55	.48	.81
MR Egger	OR	0.97	0.02	1.25	0.001
	95% CI	0.04–88.40	0.01–17.07	0.01–11.5	0.001–0.06
	*P* value	.49	.28	.95	.59
Simple mode	OR	0.78	0.72	0.15	1.015
	95% CI	0.01–30.67	0.03–17.20	0.01–10.10	0.02–0.48
	*P* value	.49	.84	.95	.66

CI = confidence interval, IVW = inverse-variance weighted, MR = Mendelian randomization, MVPA = moderate-to-vigorous physical activity, OR = odds ratio, SNPs = single nucleotide polymorphisms, VPA = vigorous physical activity.

IVW results showed that: watching TV time (OR = 0.623, 95% CI: 0.21–1.78, *P* = .38); time spent on mobile phones (OR = 0.57, 95% CI: 0.16–1.96, *P* = .37); MVPA (OR = 0.53, 95% CI: 0.09–3.15, *P* = .49); VPA (OR = 0.62, 95% CI: 0.21–1.78, *P* = .61). There was no significant association between the above exposure factors and the risk of scoliosis. We applied the remaining 4 methods, including weighted median, MR-Egger, etc, to further confirm our findings, and Table 2 shows the complete results of the MR analysis.

To further evaluate the precision of the results, we performed sensitivity analyses, including MR-Egger intercept analysis, Cochran *Q* test, and MR-PRESSO global test. The MR-Egger intercept and MR-PRESSO analyses indicated that pleiotropy did not exist for the above exposure to scoliosis. Among the other outcomes, there was no horizontal pleiotropy because all MR-Egger intercepts were *P* > .05. There was also no heterogeneity between the results of the Cochran *Q* test and those of MR-PRESS. In addition, we performed leave-one-out analysis and plotted the above analysis. Consistently, the results showed that no single SNP had an effect on the overall causal estimate (Fig. 2).

**Figure 2.**
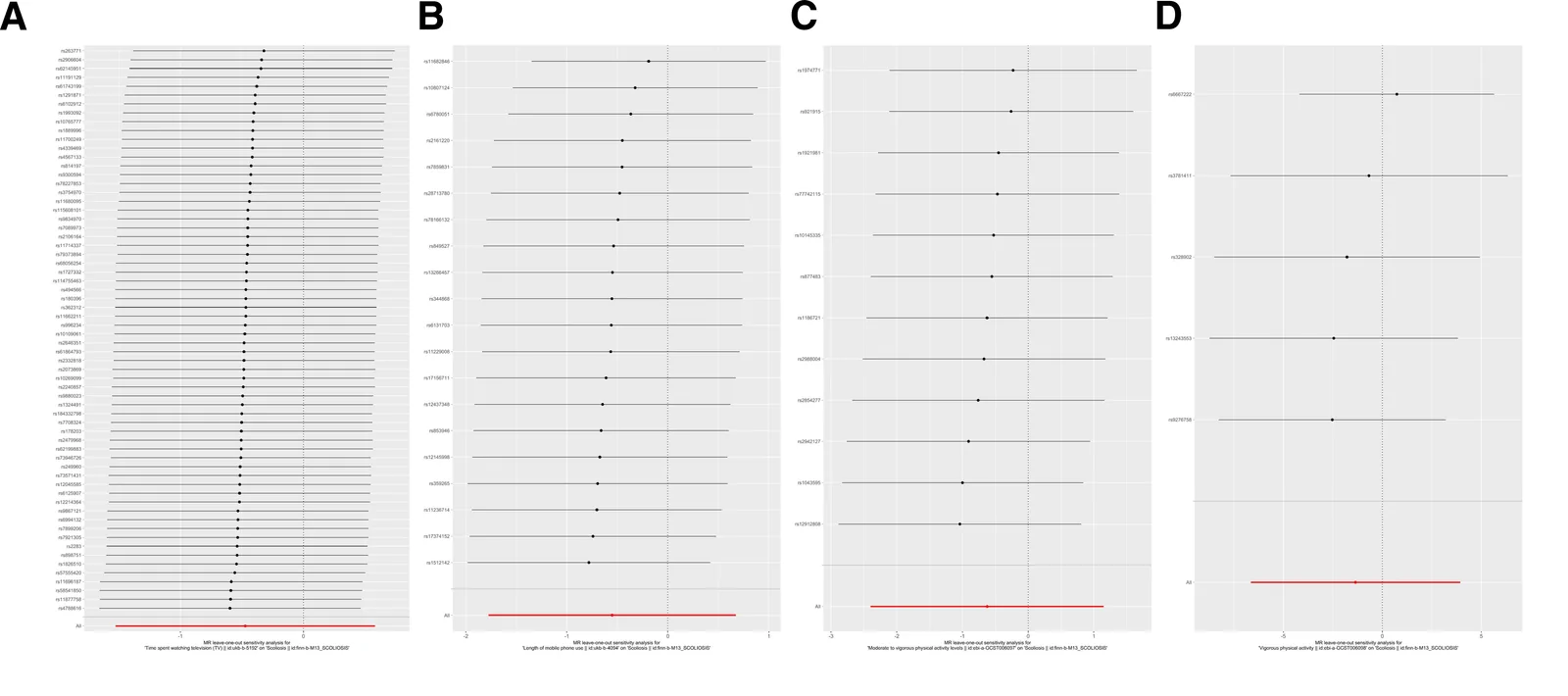
Leave-one-out sensitivity analysis. The causal estimate (with 95% confidence interval) for the association between each exposure (television watching, A; mobile phone use, B; MVPA, C; VPA, D) and scoliosis is calculated repeatedly, leaving out one single nucleotide polymorphism (SNP) at a time. The overall estimate from the inverse variance weighted (IVW) method is represented by the solid vertical line. The analysis demonstrates that no single SNP disproportionately drives the null association observed in the primary analysis. MVPA = moderate-to-vigorous physical activity, VPA = vigorous physical activity.

To further test the stability of the above results, we used funnel plots and scatter plots to visually assess horizontal pleiotropy and heterogeneity. The funnel plots showed a basically symmetrical distribution of causal effects without significant bias (Fig. 3), while scatter plots further confirmed that there was no causal association between the above exposures and scoliosis (Fig. 4). In summary, genetically predicted TV watching, mobile phone use, MVPA, and VPA were not associated with the risk of scoliosis. Sensitivity analyses did not indicate horizontal pleiotropy or significant heterogeneity, and leave-one-out analyses showed that no single SNP disproportionately drove the estimates.

**Figure 3.**
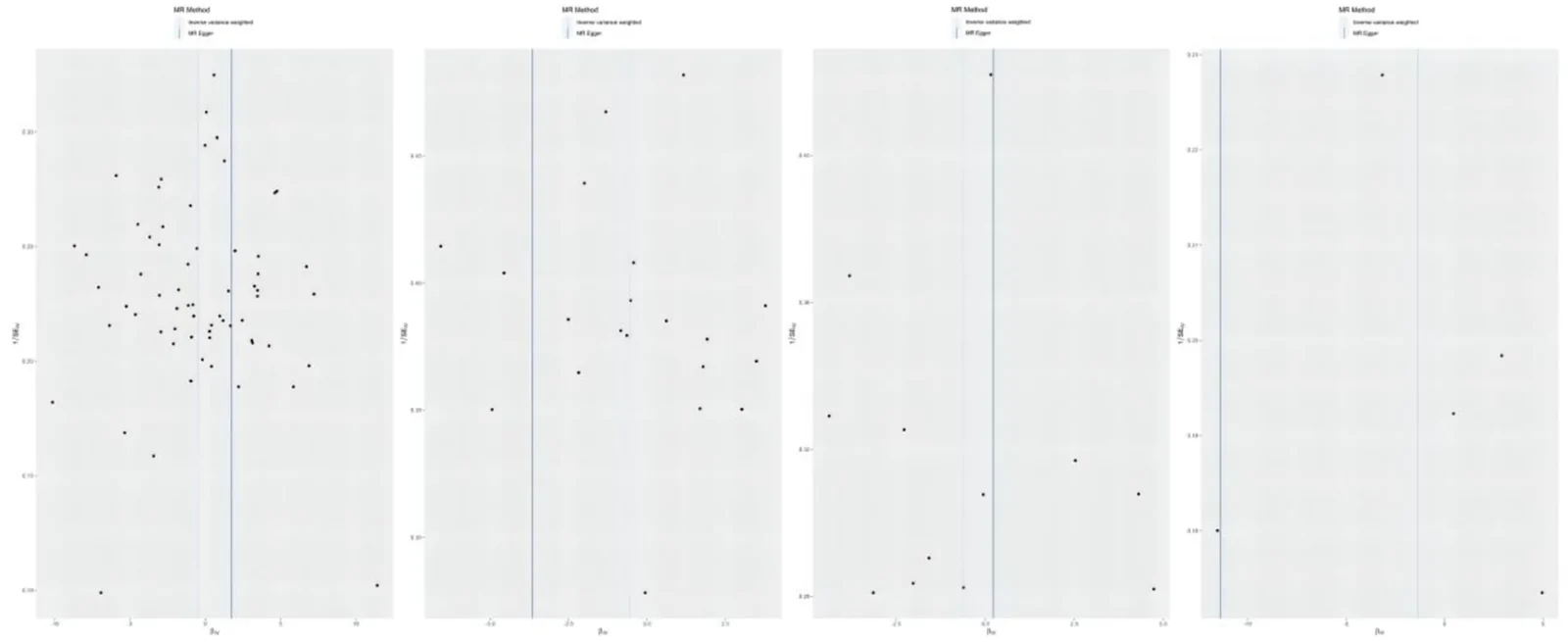
Funnel plots for assessment of directional pleiotropy. Each point represents an individual SNP’s causal estimate plotted against its precision (1/standard error). The solid vertical line indicates the pooled IVW estimate, and the dashed lines represent its 95% confidence interval. Symmetrical distribution of the SNPs around the IVW estimate suggests the absence of significant directional pleiotropy. IVW = inverse-variance weighted, SNP = single nucleotide polymorphism.

**Figure 4.**
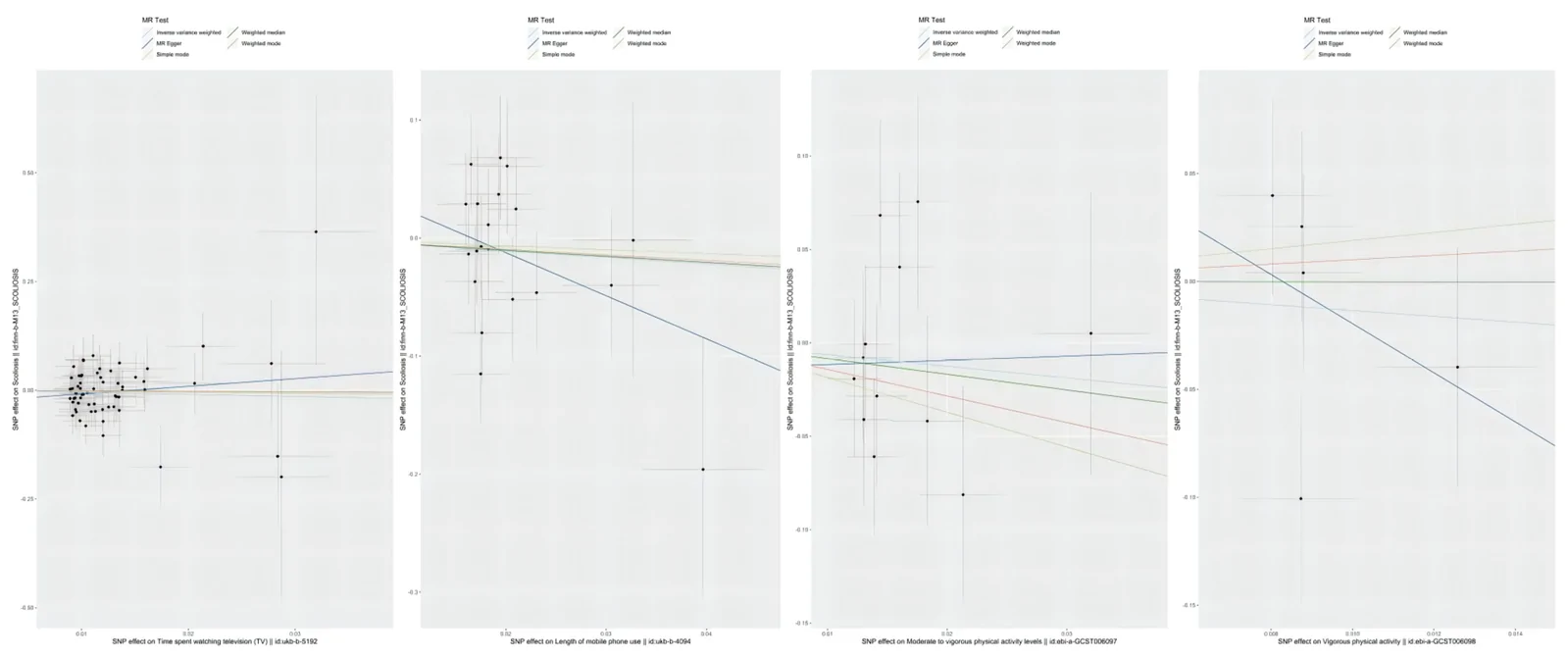
Scatter plots of the genetic associations. The genetic associations of each SNP with the exposure are plotted against its associations with scoliosis. The slopes of the regression lines from different Mendelian randomization methods (e.g., IVW, MR-Egger) represent the causal effect estimates. The near-zero slopes for all exposures are consistent with the lack of a causal effect. IVW = inverse-variance weighted, MR = Mendelian randomization, SNP = single nucleotide polymorphism.

## 4. Discussion

In the past few decades, many studies^[21–23,44–52]^ have investigated the association between leisure-time sedentary behavior, PA, and scoliosis, including cross-sectional, prospective, and randomized controlled studies. However, these studies have yielded conflicting results, and no definite conclusions can be drawn from the available data. The present study, the first MR analysis with a large sample of participants, showed no causal relationship between TV watching, mobile phone use, MVPA, VPA, and scoliosis. The results obtained by our application of different MR methods are consistent.

Some of these studies are consistent with our results.^[21,44–47]^ For example, PA was not found to be a risk factor for scoliosis in a study by Zucker et al.^[45]^ Meyer et al^[21]^ further found that adolescents who practice gymnastics may be more likely to develop scoliosis, and they attributed this association to higher joint laxity. Ghanem et al^[48]^ found that classical ballet and professional swimming increased the risk of scoliosis. In a study on the relationship between place of residence, PA, use of electronic devices, and scoliosis, urban and rural girls were compared, and it was found that girls living in rural areas had a lower incidence of scoliosis.^[49]^ We found that the above studies emphasized the association between different types of exercise, residence, and lifestyle and scoliosis, while excluding the causal relationship between PA and scoliosis. Future studies should take more consideration of the potential effects of exercise type and living environment on scoliosis.

Our MR findings, which account for confounding and reverse causality, contrast with some observational studies that reported associations between these behaviors and scoliosis.^[22,50–52]^ Among them, Glavaš et al^[51]^ conducted the most extensive study, and their study confirmed that compared with students who participated in organized sports, students who did not exercise or only engaged in recreational activities had a higher prevalence of scoliosis, and they found that different types of sports had different prevalences of scoliosis. This discrepancy highlights the likelihood that the associations observed in those studies were influenced by residual confounding or reverse causality, rather than representing causal effects. For instance, scoliosis-related postural changes or discomfort might lead to reduced PA levels (reverse causality), or unmeasured factors (e.g., underlying genetic predisposition) might confound the observed relationships. For example, in the first prospective analysis involving a total of 14,062 participants, decreased physical strength and mobility at 18 months of age were reported to be associated with an increased risk of scoliosis flares between 10 and 15 years of age, suggesting that lower PA is a risk factor for scoliosis initiation. Moreover, this study adjusted for the influence of confounding factors such as age, gender, body mass index, fat mass, or low back pain.^[23]^ This may indicate that the same genetic architecture exists between leisure-time sedentary behavior, PA, and scoliosis. However, the study did not reveal the underlying mechanism of this association, and further in-depth studies are needed to explore the risk factors and interventions for scoliosis. PA is essential for the growth and development of adolescents, while scoliosis has a significant impact on adolescents’ psychology and physiology. There is conflicting evidence on the association between sedentary behavior, PA (MVPA, VPA), and scoliosis. However, according to our MR study, there may be no causal relationship between them.

Our study has several strengths. Firstly, we applied the MR approach for the first time to examine the causal relationship between sedentary behavior, PA, and scoliosis, without potential confounding factors and reverse causality. Secondly, our data were derived from GWAS and FinnGen summary level data, and the results were consistent across applying different datasets, ensuring the reliability of our findings.

However, this two-sample MR Study still has some limitations. First, the database used in this study was based on populations of European ancestry, and it is unclear whether the results would apply to populations of non-European ancestry. Therefore, confirmation of these findings in other populations is warranted. Second, unobserved pleiotropy is a major limitation of MR studies, which may affect the conclusions that assess the associations between genetically predicted sedentary leisure-time behavior, PA, and the risk of scoliosis. Finally, and most importantly, some exposures (e.g., VPA) were determined by relatively few SNPs (number, 5), which may limit statistical power and lead to widened CIs. Thus, the absence of a statistically significant association in these analyses should be interpreted as suggestive rather than definitive evidence of the absence of a causal effect. More studies are needed to validate our findings in the future.

## 5. Conclusion

This MR study found no evidence to support a causal relationship between genetically predicted leisure sedentary behavior, PA (including MVPA and VPA), and the risk of scoliosis. The associations reported in previous observational studies are likely attributable to confounding factors or reverse causality.

## Acknowledgments

The authors are grateful to the participants of all the GWAS used in this manuscript and the investigators who made these GWAS data publicly available.

## Author contributions

**Conceptualization:** Fangjun Yang, Jiantao Wen.

**Data curation:** Jiantao Wen.

**Formal analysis:** Xiaoming Qiu, Han Liu.

**Investigation:** Xiaoming Qiu, Han Liu.

**Methodology:** Jiantao Wen.

**Project administration:** Xiaoming Qiu, Jiantao Wen.

**Supervision:** Fangjun Yang, Xiaoming Qiu.

**Validation:** Fangjun Yang.

**Visualization:** Fangjun Yang, Xiaoming Qiu.

**Writing – original draft:** Fangjun Yang, Xiaoming Qiu.

**Writing – review & editing:** Fangjun Yang, Jiantao Wen.
